# Identification of distinct circulating exosomes in Parkinson's disease

**DOI:** 10.1002/acn3.175

**Published:** 2015-02-06

**Authors:** Paul R Tomlinson, Ying Zheng, Roman Fischer, Ronny Heidasch, Chris Gardiner, Samuel Evetts, Michele Hu, Richard Wade-Martins, Martin R Turner, John Morris, Kevin Talbot, Benedikt M Kessler, George K Tofaris

**Affiliations:** 1 Nuffield Department of Clinical Neurosciences, University of OxfordOxford, United Kingdom; 2 Nuffield Department of Medicine, Target Discovery Institute, University of OxfordOxford, United Kingdom; 3 Nuffield Department of Obstetrics and Gynaecology, University of OxfordOxford, United Kingdom; 4 Department of Physiology, Anatomy and Genetics, University of OxfordOxford, United Kingdom; 5 Oxford Parkinson's Disease Centre, University of OxfordOxford, United Kingdom

## Abstract

**Objective:**

Whether circulating microvesicles convey bioactive signals in neurodegenerative diseases remains currently unknown. In this study, we investigated the biochemical composition and biological function of exosomes isolated from sera of patients with Parkinson's disease (PD).

**Methods:**

Proteomic analysis was performed on microvesicle preparations from grouped samples of patients with genetic and sporadic forms of PD, amyotrophic lateral sclerosis, and healthy subjects. Nanoparticle-tracking analysis was used to assess the number and size of exosomes between patient groups. To interrogate their biological effect, microvesicles were added to primary rat cortical neurons subjected to either nutrient deprivation or sodium arsenite.

**Results:**

Among 1033 proteins identified, 23 exosome-associated proteins were differentially abundant in PD, including the regulator of exosome biogenesis syntenin 1. These protein changes were detected despite similar exosome numbers across groups suggesting that they may reflect exosome subpopulations with distinct functions. Accordingly, we showed in models of neuronal stress that Parkinson's-derived microvesicles have a protective effect.

**Interpretation:**

Collectively, these data suggest for the first time that immunophenotyping of circulating exosome subpopulations in PD may lead to a better understanding of the systemic response to neurodegeneration and the development of novel therapeutics.

## Introduction

Parkinson's disease (PD) is an age-related, neurodegenerative disease, characterized primarily by a movement disorder and pathologically by intraneuronal accumulation and misfolding of *α*-synuclein.^1^ It is now well established that clinical and pathological changes are also seen peripherally such as in the gut, and may predate central neurodegeneration.^2^ Similarly, molecular adaptations have been described in the blood of patients with PD^3^,^4^ but the significance of such nonneuronal changes for the disease process remains unclear.

Circulating microvesicles are membrane-bound nanoparticles that are released in biofluids by most cell types and depending on their cargo and site of origin, are regulated by distinct intracellular stimuli. Exosomes are 40–150 nm vesicles derived from the invagination of the limiting membrane of late endosomes, whereas ectosomes are 100–1000 nm in size and originate from the plasma membrane.^5^ Because of this mode of generation, microvesicles may reflect intracellular changes that occur in response to a pathological condition or represent a form of paracrine interaction between healthy and disease tissues. Such interactions have been extensively documented in cancer,^6^ including brain tumors,^7^ where microvesicles may convey either antineoplastic or trophic support to the invading malignant cells. Given that PD has widespread extracranial manifestations, we asked whether circulating microvesicles derived from patients’ sera exhibit distinct biochemical composition and function.

## Materials and Methods

### Patient samples

Serum samples were obtained from PD patients and age-matched controls that were enrolled in the Oxford PD Cohort. PD patients diagnosed within the previous 3.5 years were prospectively recruited over 2 years (ethics study 10/H0505/71). For proteomic analysis, the control subjects consisted of three groups of 12 subjects each (average age 64 years; 18 males; 18 females); the sporadic PD group consisted of three groups of 12 subjects each (average age 64 years; 17 males; 19 females); the glucocerebrosidase (GBA) heterozygous subjects consisted of one group of 13 patients (average age 62 years; seven males; six females). As a disease-control group, patients with amyotrophic lateral sclerosis (ALS) were obtained from the Oxford Study for Biomarkers in Motor Neuron Disease (“BioMOx”, ethics study 08/H0605/85). Serum was used from 22 individual patients (average age 65 years; 14 males; eight females). For the validation phase of the study, serum from 90 different subjects was used as follows: controls consisted of three groups of 10 subjects each (average age 68 years; nine males; 21 females). PD consisted of six groups of 10 subjects each: three groups comprised PD patients at Hoehn & Yahr stage 1 (average age 64 years; 20 males; 10 females) and three groups consisting of PD patients at Hoehn & Yahr stage 2 (average age 67 years; 18 males; 12 females). For proteomics, 0.6 mL of serum was pooled from each individual in each group. For cell-based assays, 1 mL of serum was individually extracted from 30 patients and 30 age-matched controls.

### Microvesicle isolation from serum

Serum samples were subjected to serial centrifugation with all steps performed at 4°C: initially at 800*g* for 10 min followed by 1500*g* for 10 min and lastly 17,000*g* for 15 min. The resultant supernatant was filtered (0.2 *μ*m filter) and spun at 160,000 g for 1 h. The pellet from each group was resuspended in 5 mL of Hanks Balanced Salt Solution (HBSS) and spun in an ultracentrifuge at 160,000 g for 1 h at 4°C. The final pellet containing washed microvesicles was resuspended in HBSS for subsequent analysis.

### Nanoparticle-tracking analysis

Microvesicle size and concentration were assessed using a NS500 instrument (Nanosight Ltd., Amesbury, UK) equipped with a 405-nm laser and a CMOS camera as described previously.^8^

### Mass spectrometry

Samples were processed by nano-UPLC-MS/MS using a Thermo LTQ Orbitrap Velos mass spectrometer coupled to a Waters nanoAcquity UPLC system as described previously^9^ with some modifications. In brief, proteins were precipitated using chloroform/methanol followed by trypsin digestion, desalting using C18 SepPac cartridges (Waters, Milford, MA, USA) and resuspended in water with 0.1% Trifluoroacetic acid, 2% Acetonitrile. Samples were subsequently analyzed by nanoUPLC-MS/MS using a Waters, nanoAcquity column, 75 *μ*m × 250 mm, 1.7 *μ*m particle size, and a gradient of 1–40% acetonitrile in 60 min at a flow rate of 250 nL/min. Mass spectrometry analysis was performed on a Thermo LTQ Orbitrap Velos (60,000 Resolution, Top 20, CID, Waltham, MA, USA). Proteins were identified using PEAKS (http://www.bioinfor.com/peaks) by applying a false discovery rate (FDR) of 1% and quantified with LC Progenesis softwarev v4.1 (Nonlinear Dynamics, Newcastle, UK). Principal component analysis (PCA) plots were generated using XLSTAT (Addinsoft Inc., New York, USA) & Microsoft Excel (Microsoft, Redmond, WA, USA). Gene Ontology term enrichment analysis was conducted with AmiGO2/Panther using the 10 most enriched GO terms (*P*-value) in the list of identified proteins from each sample.

### Immunoblotting

Samples were run on NuPAGE 10–12% Bis-Tris gels (Life Technologies, Paisley, UK). The following primary antibodies were used: rabbit anti-flotillin (1:1000 Abcam, Cambridge, UK), rabbit anti-Tsg101 (Abcam 1:250), rabbit anti-syntenin 1 (Abcam, 1:1000). Blots were visualized using HRP-conjugated secondary antibodies and the ECL Detection Reagent (GE Healthcare, Little Chalfont, UK).

### Preparation and treatment of cortical neurons

All experiments were conducted in accordance with institutional and governmental guidelines. Primary cortical neurons were prepared from P0 rats. Cortical neurons (1.5 × 10^5^ cells/24-well or 4 × 10^4^/96-well) were cultured for 7 days in neurobasal/B27 medium at 37°C. Half of the medium was replaced every 3 days and mitotic inhibitor (2 *μ*mol/L cytosine *β*-D-arabinofuranoside [araC], Sigma (St Louis, MO, USA) was added during the first media exchange to arrest glial growth. For nutrient deprivation (ND), neurons were cultured for 5 h in medium lacking B27 supplement before exposed to microvesicles or liposomes. Natural or synthetic vesicles were applied to ND medium and incubated for further 16 h. The number of microvesicles and liposomes was determined by nanoparticle-tracking analysis (NTA) analysis and equal numbers of vesicles were added (800 vesicles/neuron, 24-well; 80 vesicles/neuron, 96-well) as per previously published protocols.^10^ Liposomes (Sigma) were prepared by rotatory evaporation and resuspended in HBSS. For sodium arsenite experiments, neurons were cultured in neurobasal/B27 medium. Neurons preincubated with microvesicles were treated with 0.5 mmol/L arsenite for 1 h at 37°C.

### Immunofluorescence staining of cortical neurons

Cortical neurons were plated, fixed, and stained with mouse anti-*β*-III tubulin (1:1000, BioLegend, Dedham, MA, USA), rabbit anti-cleaved caspase 3 (1:400, CellSignaling Technology, Danvers, MA, USA), and rabbit anti-syntenin 1 (Abcam, 1:2000). Goat anti-mouse AlexaFluor488 and goat anti-rabbit AlexaFluor568 (both Invitrogen, 1:500) were used as secondary antibodies and cells were imaged with a fluorescent (DM2500, Leica, Solms, Germany) or confocal (LMS710, Zeiss, Oberkochen, Germany) microscope. For quantification of caspase three positive neurons, digital photographs of five random fields were taken (Rotera XR Fast 1394; QImaging, Surrey, UK) and the percentage of caspase three positive, *β*-III tubulin-positive neurons were counted in an unbiased manner. All experiments were repeated three times and presented data were based on a total count of at least 2500 neurons per condition per experiment.

### Microvesicle uptake studies

Purified patient microvesicles were labeled with the fluorescent marker PKH67 (Sigma) for 3 min at 37°C followed by ultracentrifugation at 160,000*g* for 1 h. Labeled microvesicles were washed once and immediately added to neurons for 4–12 h. Uptake was visualized by confocal microscopy.

### Neuronal viability assays

Neuronal viability was assayed by MTT assay: 0.5 mg/mL 3-(4,5-dimethylthiazol-2-yl)-2,5-diphenyltetrazoliumbromide (MTT, Sigma) was dissolved in ND medium and added to cortical neurons for 2 h. Formazan crystals were solubilized in Dimethyl sulfoxide and absorbance was measured at 570 nm using a plate reader (FLUOstar Optima; BMG Labtech, Ortenberg, Germany).

### Statistical analysis

Raw protein abundance values were first normalized to total protein content. Label-free relative protein quantitation was conducted with Progenesis LCMS v4.1 (nonlinear Dynamics) on proteins identified with at least two unique peptides using one-way analysis of variance, *P* value <0.05. Microvesicle-treated neurons were compared between patients and healthy controls using *t*-test. Only *P* values < 0.05 were considered statistically significant and are indicated by asterisks. Error bars represent the standard error of the mean.

## Results

**Figure 1:**
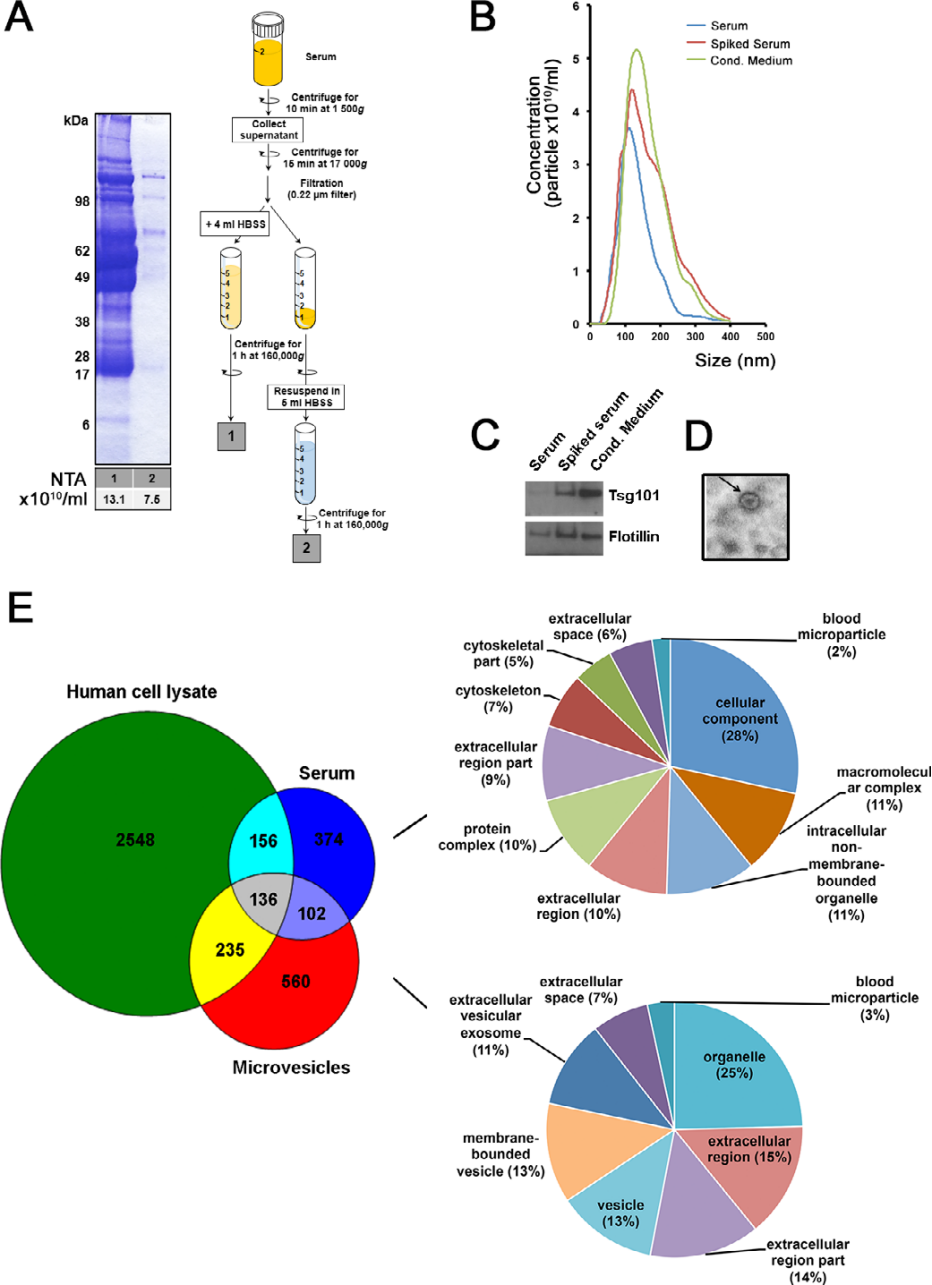
Isolation and characterization of circulating microvesicles. (A) Flow diagram showing the extraction method used and Coomassie staining of the final microvesicle preparation. Note that an additional wash step significantly reduces the number of contaminant proteins without major changes in microvesicle numbers. (B) Nanoparticle-tracking analysis of serum microvesicles revealed a major peak at the size corresponding to exosomes isolated from NSC34-conditioned media, whereas spike-in experiments showed a further increment in microvesicles of the same size. (C) Immunoblotting confirmed the presence of the exosome markers flotillin1 and Tsg101 both in cell-conditioned media and serum preparations (D) Electron microscopy showed membrane-bound microvesicles, negatively stained with 2% aqueous uranyl acetate on a formvar film. (E) Venn diagram showing protein overlap between albumin/immunoglobulin depleted serum, serum microvesicles, and human cell lysates. Proteins were quantified using Oribtrap-Velos LC-MS/MS. The enrichment for serum-derived microvesicles enables the identification and quantitation of proteins that are not detected in routinely processed serum samples as shown by gene ontology analysis.

We successfully adapted previously validated protocols based on filtration and differential centrifugation,^11^ to isolate circulating microvesicles from human serum. As shown in Figure1A, our protocol effectively minimized contamination with highly abundant serum proteins, thus overcoming a major challenge in the analysis of complex proteomes. We used NTA to quantitate the number of purified microvesicles and found that the mean size of the most abundant vesicles (about 120 nm) corresponded to exosomes. This conclusion was supported by the detection of the common exosomal markers Tsg101 and flotillin by immunoblotting and electron microscopy showing that the isolated microvesicles are membrane bound (Fig.1B–D). As a positive control, we spiked the serum with purified exosomes from conditioned media derived from NSC34 cells. We observed an increment in the corresponding vesicle size and abundance indicative of exosomes as well as an increase in relevant protein markers (Fig.1B and C).

We then asked whether the proteomic profile of circulating microvesicles differed in PD compared to controls. In pilot experiments, we determined that at least 7 mL of a starting volume of serum was needed for adequate protein detection in the final microvesicle preparation. Since this volume is not easily accessible in large cohorts of patients, we performed this initial study on grouped serum samples as detailed in the Methods section. One advantage of this approach, which has been used widely in serum and plasma proteomic studies, especially investigations of disease mechanisms^12^ is that it may minimize biological variation, making it more likely to detect robust biochemical trends that are relevant to the disease process. To assess the sensitivity of our mass spectrometric analysis (Orbitrap LC-MS/MS) on these preparations, we first compared the abundance of proteins identified in grouped sera of the same 12 healthy volunteers when extracted by either a standard albumin/immunoglobulin depletion method^13^ or using our isolation protocol. We found that our method of microvesicle isolation identified a group of proteins that were not detected among the most abundant proteins in routinely processed sera (Venn diagram, Fig.1E). In addition, gene ontology term enrichment analysis confirmed that exosome preparations had a distinct profile with 40% of identified proteins being vesicle-associated (Fig.1E). Thus, pooled sera could be used to identify unique biochemical trends that are enriched in circulating microvesicles from disease and healthy-controlled conditions.

**Figure 2:**
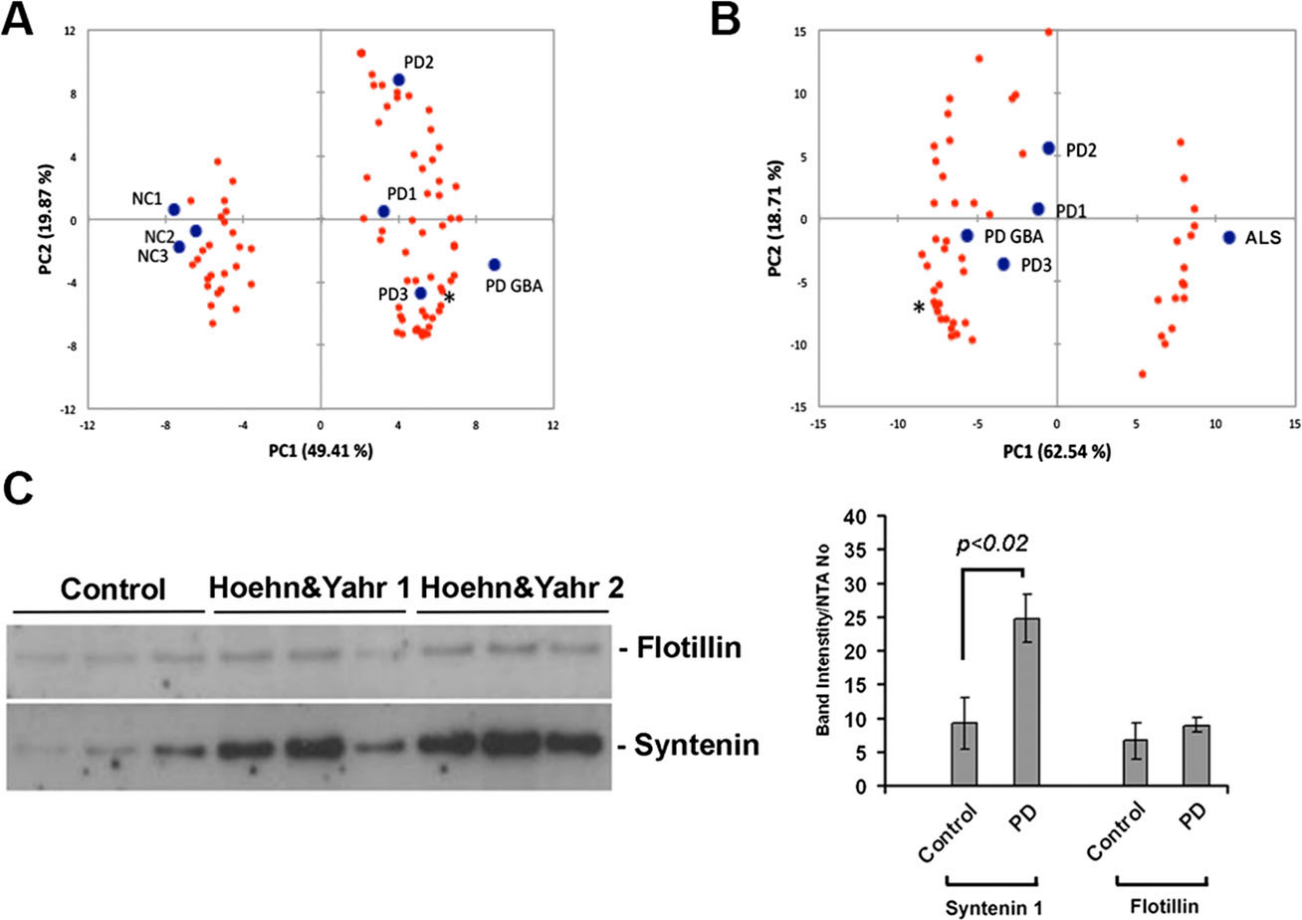
Differential proteomic composition of PD-derived microvesicles. (A) Significant protein differences were calculated after grouping of the samples into controls and PD (ANOVA *P* < 0.05). Principal component analysis (PCA) shows the separation of the analyzed samples (blue dots) and the loading of the 82 proteins (red dots). Syntenin 1 is marked with * (1.8-fold increase, *P* < 0.009). (B) Fifty-three proteins of the 82 proteins in (A) further separate PD from ALS samples (ANOVA *P* < 0.05). (C) Immunoblotting with specific antibodies against syntenin 1 confirmed the mass spectrometry finding in a separate group of early PD patients (Hoehn & Yahr 1 or 2) compared to controls (*P <* *0.02, *t*-test). PD, Parkinson's disease; ANOVA, analysis of variance; ALS, amyotrophic lateral sclerosis.

We then performed the same mass spectrometric analysis on grouped patient samples. We used three biological replicates for sporadic PD-, age-, and comorbidity-matched controls and one group of patients with ALS, an unrelated neurodegenerative disease. Because PD is heterogeneous, we also considered whether changes that are detected in sporadic disease were also seen in patients with heterozygous mutations in GBA, which causes PD that is clinically and pathologically indistinguishable from sporadic cases. Protein identification was based on unique peptides identified in two technical replications. Using this cutoff, we detected 1033 (Table S1) proteins across all patient groups with a FDR of less than 1%. Label-free relative protein quantitation was conducted with Progenesis LCMS v4.1 (nonlinear Dynamics) on proteins identified with at least two unique peptides. Eighty-two proteins were differentially abundant between control and PD groups (Fig.2A and Table S2). It is noteworthy that the four PD groups including the one with GBA mutations (blue dots representing the average vectorial position of all proteins in each sample, Fig.2A) clustered distinctly from control or ALS samples. These data suggest that GBA-positive and sporadic PD samples contain similar microvesicle-associated protein changes. Based on these data, we found that 54 of the 82 proteins further differentiated PD from ALS-derived microvesicles (Fig.2B, and Tables S3, S4) with 23 of these 54 proteins previously shown to be constituents of exosomes (ExoCarta dataset).^14^ Notably, we did not detect enrichment for disease-associated proteins such as *α*-synuclein or tau in these preparations. To confirm the result of our proteomic analysis, we then tested by immunoblotting the abundance of one of these exosomal markers, syntenin 1, in samples prepared from a different set of grouped patient samples (total 90 different subjects divided into groups of 10). We found that syntenin 1 was elevated in cases of early PD (Hoehn and Yahr 1 and 2) compared to control sera when corrected for either total vesicle number or the exosomal protein flotillin (Fig.2C). Collectively, our proteomic profiling indicates that circulating exosomal cargoes within the microvesicle preparations are differentially regulated in neurodegenerative diseases.

**Figure 3:**
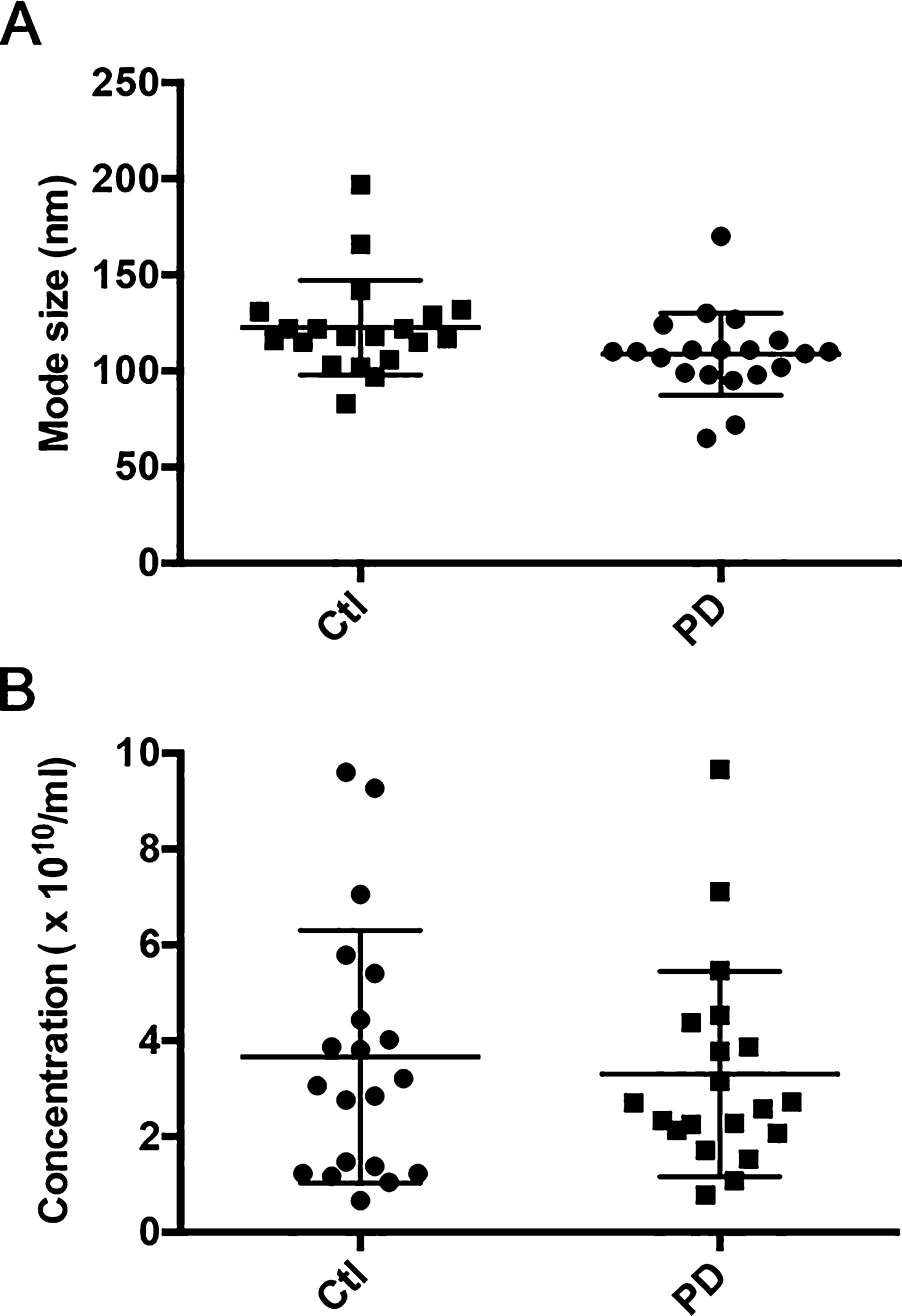
Microvesicle abundance and size do not differ between PD patients and controls. NTA was performed on microvesicles extracted from individual PD patient samples and healthy-matched controls (*n* = 20 per group). The NTA profiles showed a single peak that is typical of exosomes (100–120 nm) as demonstrated by the close mode size clustering of microvesicles from each sample (A). When averaged, the mean size (panel A) or concentration (B) of exosomes was not significantly different between the two groups. Error bars indicate standard deviation. PD, Parkinson's disease; NTA, nanoparticle-tracking analysis.

**Figure 4:**
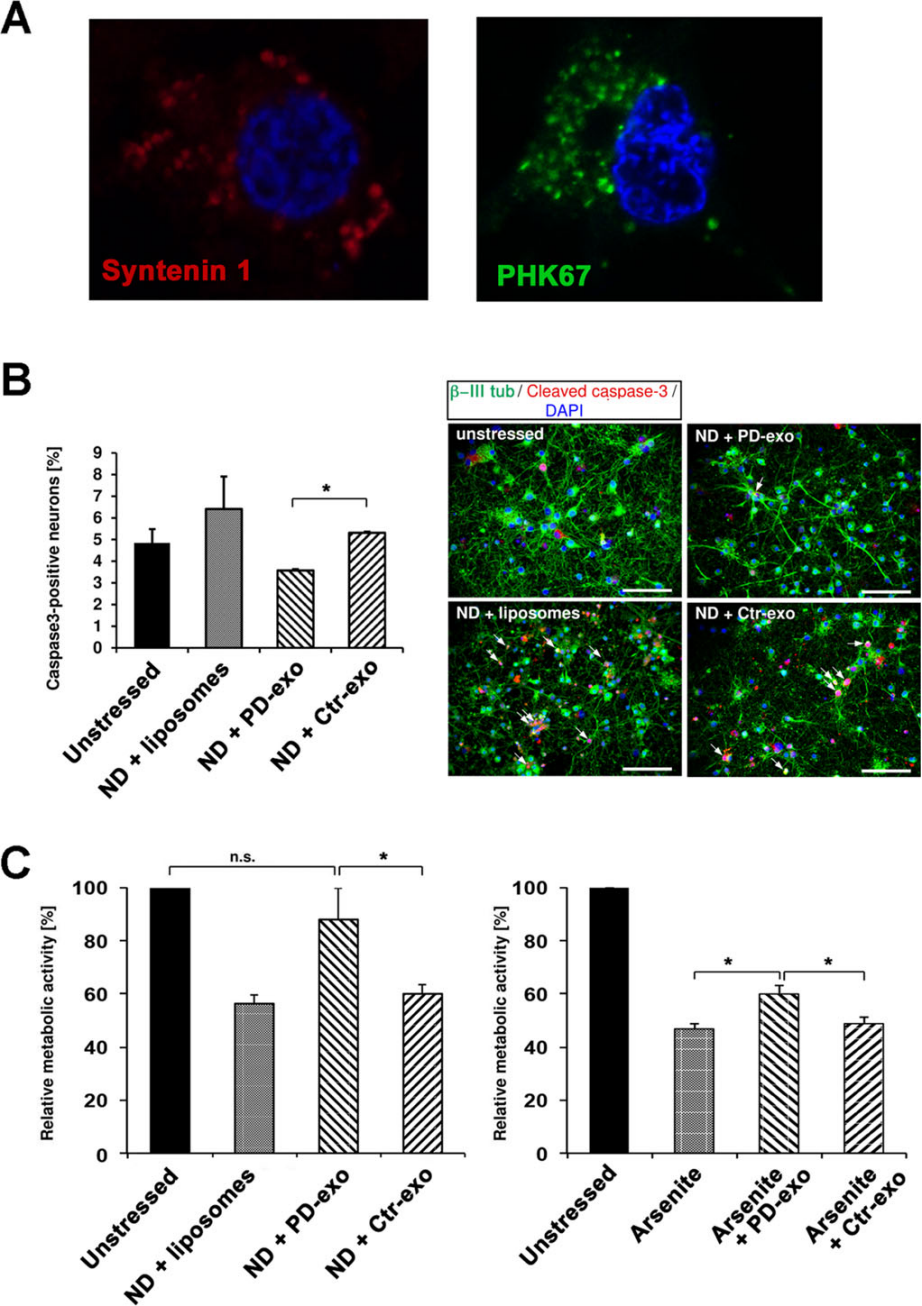
Parkinson's disease (PD)-derived microvesicles convey a neuroprotective effect. (A) Confocal images of human-specific anti-syntenin 1 (red) and 4‘,6-diamidino-2-phenylindole (nucleus, blue) staining showing uptake of human microvesicles in primary rat cortical neurons. Neuronal uptake was confirmed using exosomes prelabeled with the PHK67 fluorescent dye. (B) Caspase 3 activation was significantly reduced in neurons treated with PD-derived microvesicles compared to controls (*n* = 20 per group, *P* < 0.05). Representative images of caspase 3 and *β*-III tubulin doubly positive neurons under different experimental conditions. (C) Neuronal metabolic activity was assessed by the MTT assay in neurons pretreated with PD or control exosomes under conditions of either nutrient deprivation (left graph, *n* = 30 per group, *P *<* *0.05) or treatment with 0.5 mmol/L of sodium arsenite (right graph, *n* = 20 per group, *P *<* *0.05).

Syntenin 1 is an important regulator of exosome biogenesis.^15^ Its enrichment in PD-derived samples prompted us to investigate whether the abundance of exosomes is increased in PD using NTA in individually extracted patient samples. These data showed that there is no significant difference in the number or mode size of microvesicles between PD patients and healthy controls (Fig.3). It is thus possible that enrichment of specific proteins in PD-derived exosomes may reflect subpopulations with distinct biological properties. To investigate this hypothesis, we examined the effect of microvesicles extracted from individual patients on primary rat cortical neurons subjected to ND, which was previously shown to induce both exosome uptake and oxidative stress.^10^ The identification of syntenin 1 enabled us to study whether primary rat cortical neurons internalize human exosomes. To this end, we used human-specific antibodies and observed syntenin 1-positive vesicles in neurons treated with patient-derived microvesicles (Fig.4A) but not liposomes or untreated controls (not shown). Using microvesicles prelabeled with the fluorescent dye PKH67, we confirmed that these vesicles are internalized rather than fused with the plasma membrane (Fig.4A). We then compared the effect of PD-derived microvesicles to healthy controls. We first stained and quantified in an unbiased fashion microvesicle-treated *β*-III tubulin-positive neurons for caspase 3 activation, which is an indicator of apoptosis. Strikingly, we found that neurons treated with PD-derived microvesicles had significantly less activated caspase 3-positive neurons compared to those treated with control microvesicles (Fig.4B). To corroborate these findings using an alternative readout of toxicity, we used the MTT assay in either nutrient deprived or sodium arsenite treated neurons that were preincubated with microvesicles. We found that the metabolic activity of neurons treated with PD-derived microvesicles was significantly improved under both conditions when compared to ones derived from age-matched controls (Fig.4C).

## Discussion

Our data provide novel insights into the disease-specific systemic response that is induced in neurodegenerative disorders. The discovery of distinct protein changes in the absence of significant differences in microvesicle numbers or size suggests that in PD there is a subpopulation of vesicles that is either differentially regulated or enriched for certain proteins in response to the disease process. Evidence from proteomics, imaging and nanoparticle-tracking analysis indicate that the majority of these microvesicles have properties that are typical of exosomes. It is therefore likely that disease-relevant exosomes are detected in the circulation and serve-specific functions. This conclusion is also supported by the differential effect of PD-derived exosomes using our viability readouts in neuronal cultures. Although these neuronal assays are not disease models, they raise for the first time the intriguing possibility that there is an innate systemic response to neurodegeneration that may be protective. Whether this is tissue-specific or generalized remains currently unknown, but evidence suggests that peripherally injected exosomes can cross the blood–brain barrier in mice to exert intraneuronal effects.^16^ On the other hand, exosomes have been proposed as effectors of the prion-like propagation of misfolded proteins.^17^ Contrary to a previous report that *α*-synuclein is increased in plasma microvesicles from PD patients,^18^ our unbiased grouped analysis did not detect enrichment of *α*-synuclein in PD-derived serum microvesicles or a detrimental effect when taken up by neurons. It is possible that such discrepancies may reflect technical differences in microvesicle isolation but given that *α*-synuclein is abundant in the circulation and known to easily associate with membranes, caution is required when interpreting candidate-based measurements. Accordingly, *α*-synuclein was not detected in a proteomic analysis of pooled CSF exosomes.^19^ It is important to note that our data do not exclude the possibility that *α*-synuclein is secreted in exosomes in the neuronal microenvironment under pathological conditions.

Despite the unique protein changes in PD-derived microvesicles reported herein, the specific mediators of their biological functions and regulators of their release remain unclear. Our identification of proteins with chaperone or anti-oxidant properties (Table S3) suggests two potential protective effectors. Exosomes are also a rich source of RNA,^20^ which could contribute to the observed or additional effects. In addition, syntenin 1 which is implicated in the biogenesis of exosomes^15^ was shown to act as an intracellular adaptor for diverse signaling pathways.^21^ An alternative explanation for our cellular observations is the differential expression of proteins in exosomes that promote specifically their neuronal uptake rather than their intracellular effect. Exosome preparations from cell lines have different cell-binding specificities when incubated with primary neurons^22^ and various membrane-bound proteins have been implicated in exosomal uptake.^23^ In our PD preparations, we found an enrichment in Integrin *β*1, whereas the common exosomal tetraspanins were equally detected in all groups. These data suggest a potential role for Integrin *β*1 in such a neuron-specific exosome binding or uptake mechanism. It is now critical to clarify these outstanding questions. In this respect, our proteomic analysis could provide useful markers for in-depth profiling of specific exosome subpopulations.

Although this study was not designed to identify biomarkers, our data suggest that immunophenotyping of circulating exosomes may hold promise for the development of tractable disease markers in neurodegenerative diseases. Importantly, further characterization of the biogenesis of selective exosome subpopulations may provide new insights into the systemic self-defense mechanisms that are activated during Parkinson's pathogenesis.

## Supporting Information

Table S1. List of the 1033 proteins that were identified across samples.

Table S2. List of proteins that were significantly up- or downregulated in PD-derived microvesicles compared to age-matched healthy controls.

Table S3. List of proteins that were differentially regulated in PD-derived microvesicles compared to those with ALS. Those previously associated with exosomes are shown in bold.

Table S4 List of proteins that were differentially regulated in ALS-derived microvesicles compared to controls.
